# Change in H^+^ Transport across Thylakoid Membrane as Potential Mechanism of 14.3 Hz Magnetic Field Impact on Photosynthetic Light Reactions in Seedlings of Wheat (*Triticum aestivum* L.)

**DOI:** 10.3390/plants10102207

**Published:** 2021-10-18

**Authors:** Ekaterina Sukhova, Ekaterina Gromova, Lyubov Yudina, Anastasiia Kior, Yana Vetrova, Nikolay Ilin, Evgeny Mareev, Vladimir Vodeneev, Vladimir Sukhov

**Affiliations:** 1 Department of Biophysics, N.I. Lobachevsky State University of Nizhny Novgorod, 603950 Nizhny Novgorod, Russia; n.catherine@inbox.ru (E.S.); kater333@inbox.ru (E.G.); lyubovsurova@mail.ru (L.Y.); nastay2903@bk.ru (A.K.); yanariya371@mail.ru (Y.V.); v.vodeneev@mail.ru (V.V.); 2 Earth’s Electromagnetic Environment Laboratory, Institute of Applied Physics of Russian Academy of Sciences, 603600 Nizhny Novgorod, Russia; ilyin@appl.sci-nnov.ru (N.I.); evgeny.mareev@gmail.com (E.M.)

**Keywords:** extremely low-frequency magnetic fields, schumann resonance frequencies, photosynthetic light reactions, proton transport, thylakoid membrane, simulation, plants, wheat

## Abstract

Natural and artificial extremely low-frequency magnetic fields (ELFMFs) are important factors influencing physiological processes in living organisms including terrestrial plants. Earlier, it was experimentally shown that short-term and long-term treatments by ELFMFs with Schumann resonance frequencies (7.8, 14.3, and 20.8 Hz) influenced parameters of photosynthetic light reactions in wheat leaves. The current work is devoted to an analysis of potential ways of this ELFMF influence on the light reactions. Only a short-term wheat treatment by 14.3 Hz ELFMF was used in the analysis. First, it was experimentally shown that ELFMF-induced changes (an increase in the effective quantum yield of photosystem II, a decrease in the non-photochemical quenching of chlorophyll fluorescence, a decrease in time of changes in these parameters, etc.) were observed under the action of ELFMF with widely ranging magnitudes (from 3 to 180 µT). In contrast, the potential quantum yield of photosystem II and time of relaxation of the energy-dependent component of the non-photochemical quenching were not significantly influenced by ELFMF. Second, it was shown that the ELFMF treatment decreased the proton gradient across the thylakoid membrane. In contrast, the H^+^ conductivity increased under this treatment. Third, an analysis of the simplest mathematical model of an H^+^ transport across the thylakoid membrane, which was developed in this work, showed that changes in H^+^ fluxes related to activities of the photosynthetic electron transport chain and the H^+^-ATP synthase were not likely a mechanism of the ELFMF influence. In contrast, changes induced by an increase in an additional H^+^ flux (probably, through the proton leakage and/or through the H^+^/Ca^2+^ antiporter activity in the thylakoid membrane) were in good accordance with experimental results. Thus, we hypothesized that this increase is the mechanism of the 14.3 Hz ELFMF influence (and, maybe, influences of other low frequencies) on photosynthetic light reactions in wheat.

## 1. Introduction

Photosynthesis is a key process in plant life providing consumption of solar energy and production of biomass. It can be affected by an action of numerous environmental stressors including high-intensity visible light [1,2,3,4,5] and ultraviolet light [6], salinity [7,8,9,10,11], non-optimal temperatures [12,13,14,15,16,17], drought [16,17,18,19,20], etc.

Transfer of electric charges is an important stage of photosynthetic processes because light reactions of photosynthesis include transport of electrons and protons through the electron transport chain (ETC) [21,22,23,24,25], and ATP synthesis is based on H^+^ transport through the H^+^-ATP synthase in the plasma membrane [21,23,25]. The H^+^ transport can also indirectly influence photosynthetic processes because changes in pH in the lumen (acidification) and the stroma (alkalization) of chloroplasts regulate photosynthetic processes through the induction of the non-photochemical quenching of the chlorophyll fluorescence (NPQ), including the energy-dependent component of NPQ related to protonation of PsbS proteins in photosystem II (PSII) [4,25,26,27,28,29,30,31,32], activation of enzymes of the Calvin–Benson cycle caused by the high pH optimum of some enzymes [33,34,35], and increasing activity of the ferredoxin–NADP reductase related to pH-dependent changes in its localization in the stroma and thylakoid membrane [36,37].

Considering this influence of the stromal and lumenal pH on photosynthetic processes, photosynthesis can also depend on an additional H^+^ flux across the thylakoid membrane in the chloroplast. It can be related to a passive proton leakage across this membrane [11,38,39] and an H^+^ transport by transporters including the H^+^/Ca^2+^ antiporter [40], the H^+^/K^+^ antiporter [41,42,43], and the proton/phosphate transporters [44]. It is interesting that changes in these processes of H^+^ transport can participate in photosynthetic damage by action of stressors [16,38,39] (e.g., heating can increase proton permeability of the thylakoid membrane and, thereby, disrupts forming proton gradient across this membrane and synthesis of ATP) and in a photosynthetic adaptation to this action [41,42,43,44] (e.g., increase in proton flux caused by H^+^/K^+^ antiporter can accelerate photosynthetic adaptation to the fluctuation of light intensities through the acceleration of changes in NPQ).

Thus, the transport of electrons and protons across the thylakoid membrane plays a key role in light reactions of photosynthesis. Therefore, it can be expected that photosynthetic processes in plants may be affected by magnetic fields (MFs). There are several types of MFs in the environment: stationary geomagnetic fields, stationary artificial magnetic fields, and non-stationary MFs. Extremely low-frequency magnetic fields (ELFMFs) are an important type of non-stationary MFs [45]; they include artificial MFs with industrial frequencies (50 or 60 Hz) and natural MFs, which are mainly related to magnetospheric substorms and lightning. Observations show that the spectrum of these natural ELFMFs has maxima around the frequencies 7.8, 14.3, 20.8, 27.3, and 33.8 Hz, which are the eigenfrequencies of the Earth–ionosphere resonator and are known as Schumann resonances [45,46,47].

Several results have confirmed the influence of ELFMFs on photosynthesis and related processes (e.g., see reviews [48,49,50,51]). It was shown that ELFMFs can modify photosynthetic CO_2_ assimilation and transpiration [52,53,54], stimulate expression of the gene of the small subunit of the ribulose 1,5-bisphosphate carboxylase/oxygenase [52], and influence the content of photosynthetic pigments including an increase in the content of chlorophylls and carotenoids and an increase in the ratio between chlorophyll a and chlorophyll b concentrations [52,53,55,56]. However, only two studies [52,54] were devoted to an analysis of the influence of ELFMFs on plant seedlings; both works analyzed only MFs with the 50 Hz industrial frequency and did not analyze photosynthetic light reactions.

The problem of the influence of ELFMFs with frequencies of the Schumann resonance (7.8, 14.3, and 20.8 Hz) on parameters of photosynthetic light reactions was investigated in a previous study [57]; ELFMFs with the 18 µT intensity were only investigated. It was shown that both a short-term treatment by ELFMFs (30 min) and a chronic treatment by these MFs influenced parameters of photosynthetic light reactions in wheat seedlings; in particular, these treatments accelerated a light-induced NPQ activation, accelerated an increase in the quantum yield of PSII (Φ_PSII_) under illuminations and decreased a stationary NPQ. This effect was the strongest at 14.3 Hz (the second harmonic of the frequencies of the Schumann resonance). In contrast, the influence of ELFMFs on photosynthetic parameters of pea seedlings was weak and non-significant. We preliminarily hypothesized that the revealed photosynthetic changes can be related to changes in H^+^ fluxes across the thylakoid membrane. These changes can be caused by changes in the activity of ETC or H^+^-ATP synthase or changes in the additional H^+^ flux related to the proton leakage and H^+^ transporters in the thylakoid membrane.

The aim of this work was a further analysis of mechanisms of the ELFMF influence on photosynthetic light reactions in wheat seedlings (on the example of 14.3 Hz ELFMF because the photosynthetic changes were the most expressive in wheat at this frequency according to [57]). There were several questions that were analyzed: (i) Can the influence of ELFMF on photosynthetic light reactions be observed at a wide range of magnitudes of this magnetic field? (ii) Can ELFMF-induced changes in proton conductivity of the thylakoid membrane be experimentally shown? (iii) Can participation of H^+^ fluxes across the thylakoid membrane in the ELFMF-induced photosynthetic changes be theoretically shown?

## 2. Results

### 2.1. Influence of Treatment by 14.3 Hz ELFMF with Different Intensities on Parameters of Photosynthetic Light Reactions in Wheat Seedlings

**Figure 1:**
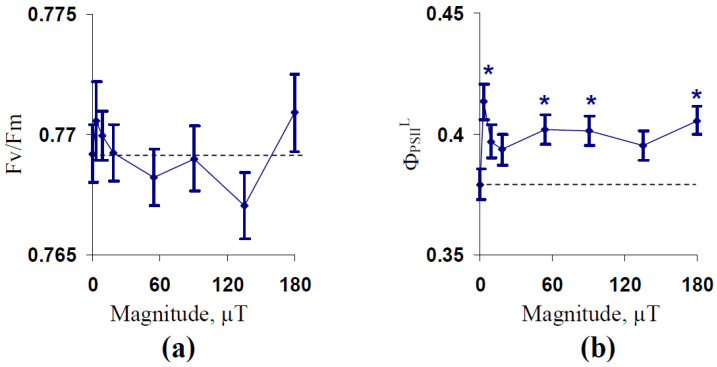
Dependencies of potential quantum yield of PSII (F_v_/F_m_) (**a**) and its effective quantum yield under illumination (Φ_PSII_^L^) (**b**) on the magnitude of extremely low-frequency magnetic field (ELFMF) with 14.3 Hz frequency in wheat seedlings (*n* = 20–30). The action of the artificial ELFMF was initiated before the dark adaptation; the 30 min treatment was used. Photosynthetic parameters were measured under the treatment. Control seedlings were not treated by the ELFMF (dotted line marked the photosynthetic parameters in these seedlings). *, the difference between photosynthetic parameters in the experimental and control wheat seedlings was significant (*p* < 0.05).

The dependence of stationary parameters of the photosystem II (PSII) on the magnitude of the 14.3 Hz ELFMF was firstly investigated. Figure 1a shows the absence of significant changes in the potential quantum yield of PSII under the treatment by the 14.3 Hz ELFMF with different magnitudes. In contrast, this ELFMF increased Φ_PSII_ under illumination (Φ_PSII_^L^) in comparison to the control value (without treatment by this MF) (Figure 1b). The effect was maximal and significant under the treatment by the 3 µT magnitude of the 14.3 Hz ELFMF. Significantly increased Φ_PSII_^L^ were observed under the treatment by ELFMF with the 54, 90, and 180 µT magnitudes. The effect was not significant under the treatment by ELFMF with the 9, 18, and 135 µT magnitudes; however, a tendency toward increased Φ_PSII_^L^ was observed in these variants. It should be noted that the result is in an accordance with a previous study [57] because this work showed that the 14.3 Hz ELFMF with 18 µT magnitude induced a non-significant increase in the Φ_PSII_^L^.

Further, we investigated the influence of the magnitude of the 14.3 Hz ELFMF on a maximal value of non-photochemical quenching (NPQ_max_), a fast-relaxing component of the non-photochemical quenching under illumination (NPQ_F_) showing the energy-dependent component of NPQ [27], and a slow-relaxing component of the non-photochemical quenching (NPQ_S_) showing long-term components of NPQ related to the “state transition” and photodamage [4,27,28].

**Figure 2:**
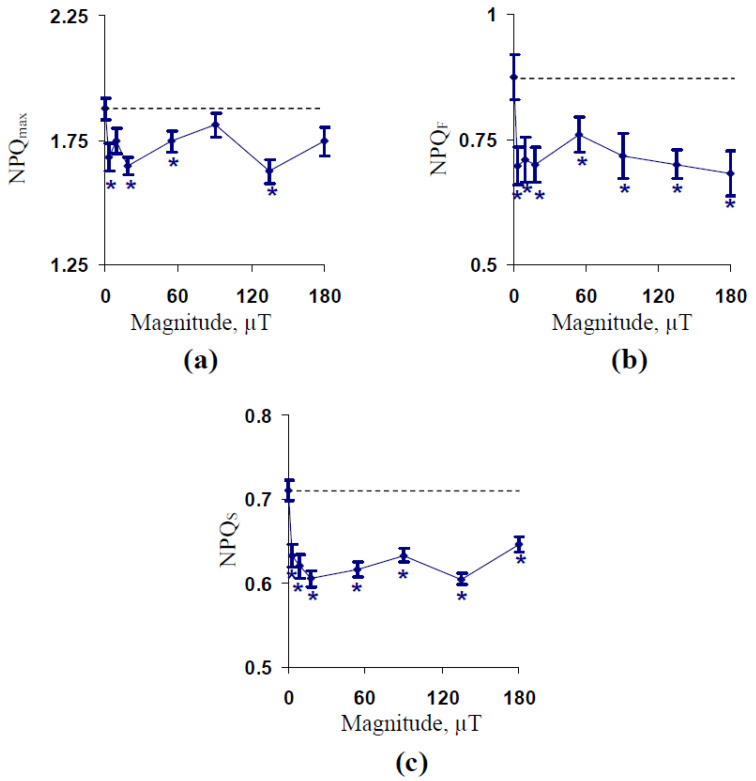
Dependencies of maximal value of non-photochemical quenching (NPQ_max_) (**a**), a fast-relaxing component of the non-photochemical quenching under illumination (NPQ_F_) (**b**), and a slow-relaxing component of the non-photochemical quenching after this illumination (NPQ_S_) (**c**) on the magnitude of ELFMF with the 14.3 Hz frequency in wheat seedlings (*n* = 20–30). The action of the artificial ELFMF was initiated before the dark adaptation; the 30 min treatment was used. Photosynthetic parameters were measured under the treatment. Control seedlings were not treated by ELFMF (dotted line marked the photosynthetic parameters in these seedlings). *, the difference between photosynthetic parameters in the experimental and control wheat seedlings was significant (*p* < 0.05).

Figure 2a shows that treatment by the 14.3 Hz ELFMF decreased NPQ_max_ in comparison to the control value. The decrease was significant under treatments with different magnitudes of ELFMF excluding the non-significant decrease under the treatment by the 14.3 Hz ELFMF with the 90 µT intensity. Analysis of dependencies of NPQ_F_ (Figure 2b) and NPQ_S_ (Figure 2c) on the magnitude of the 14.3 Hz ELFMF showed that all investigated variants of the treatment of wheat seedlings by ELFMF significantly decreased parameters of NPQ. It is interesting that the dynamics of changes in NPQ_F_ and NPQ_S_ were not completely similar (a correlation coefficient between averaged values of these parameters was 0.80).

**Figure 3:**
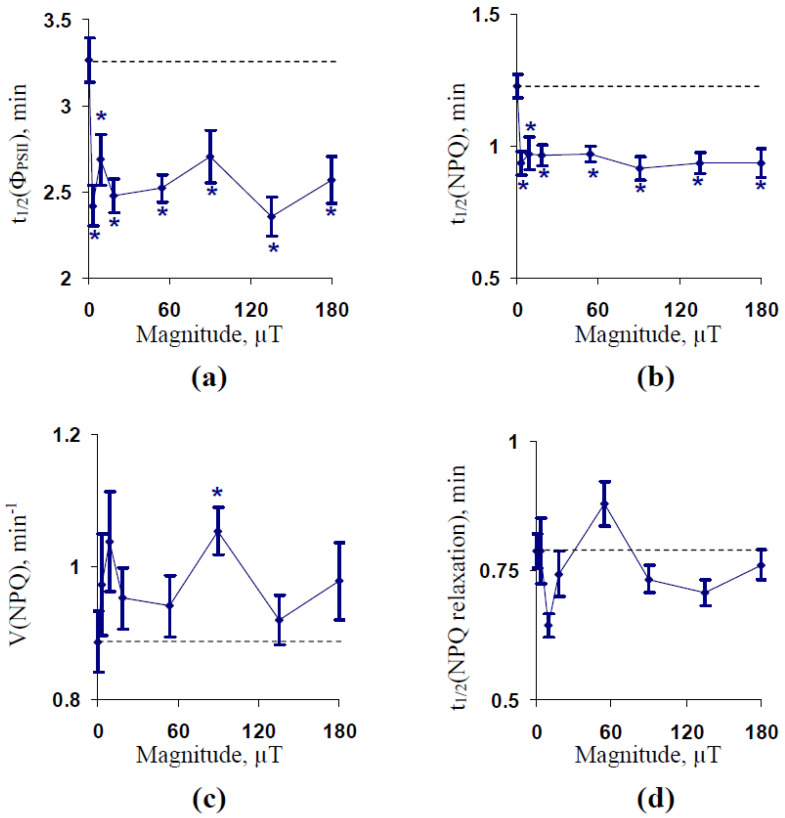
Dependencies of time taken for 50% increase in Φ_PSII_ under illumination (t_1/2_(Φ_PSII_)) (**a**), time taken for 50% increase in NPQ under illumination (t_1/2_(NPQ)) (**b**), the initial velocity of NPQ increasing (V(NPQ)) (**c**), and time taken for 50% relaxation of NPQ after the termination of illumination (t_1/2_(NPQ relaxation)) (**d**) on the magnitude of ELFMF with the 14.3 Hz frequency in wheat seedlings (*n* = 20–30). The action of the artificial ELFMF was initiated before the dark adaptation; the 30 min treatment was used. Photosynthetic parameters were measured under the treatment. Control seedlings were not treated by the ELFMF (dotted line marked the photosynthetic parameters in these seedlings). *, the difference between photosynthetic parameters in the experimental and control wheat seedlings was significant (*p* < 0.05).

Figure 3 shows ELFMF-induced changes in time taken for 50% increase in Φ_PSII_ under illumination (t_1/2_(Φ_PSII_)), time taken for 50% increase in NPQ under illumination (t_1/2_(NPQ)), the initial velocity of NPQ increasing (V(NPQ)), and time taken for 50% relaxation of NPQ after the termination of illumination (t_1/2_(NPQ relaxation)). It was shown that t_1/2_(Φ_PSII_) and t_1/2_(NPQ) were significantly decreased in comparison to the control values of these parameters under treatment by all investigated magnitudes of the 14.3 Hz ELFMF (Figure 3a,b). Changes in t_1/2_(Φ_PSII_) and t_1/2_(NPQ) were similar; the correlation coefficient between these changes was 0.89. It should be noted that the decrease in t_1/2_(Φ_PSII_) and t_1/2_(NPQ) was in good accordance with previous results [57], which showed a strong decrease in these parameters under the treatment by ELFMS with the 18 µT magnitude and the 7.8, 14.3, and 20.8 Hz frequencies.

In contrast, an ELFMF-induced stimulation of V(NPQ) was not significant for the most of investigated magnitudes (Figure 3c). Only, ELFMF with the 90 µT magnitude induced the significant stimulation of the initial linear velocity of the NPQ increase. Finally, investigated magnitudes of the 14.3 Hz ELFMF did not significantly influence t_1/2_(NPQ relaxation) (Figure 3d).

Thus, the results of the experimental analysis showed that the 14.3 Hz ELFMF can modify parameters of photosynthetic light reactions in wheat seedlings under treatment by magnetic fields with different magnitudes (from 3 µT to 180 µT). The decrease in the times taken for a 50% increase in NPQ and Φ_PSII_ under illumination seems to be the most expressive response induced by the 14.3 Hz ELFMF.

### 2.2. Analysis of the Influence of 14.3 Hz ELFMF on the Protonmotive Force, pH Gradient, and H^+^ Conductivity across the Thylakoid Membrane

In accordance with [58,59,60], parameters of the electrochromic shift (ECS) and its relaxation were used for estimation of the protonmotive force (ECS_pmf_), pH gradient (ECS_ΔpH_), and H^+^ conductivity (g_H_) across the thylakoid membrane. Relative values of ECS_pmf_ and ECS_ΔpH_ were used in the analysis. ELFMF with the 18 µT intensity was investigated.

**Figure 4:**
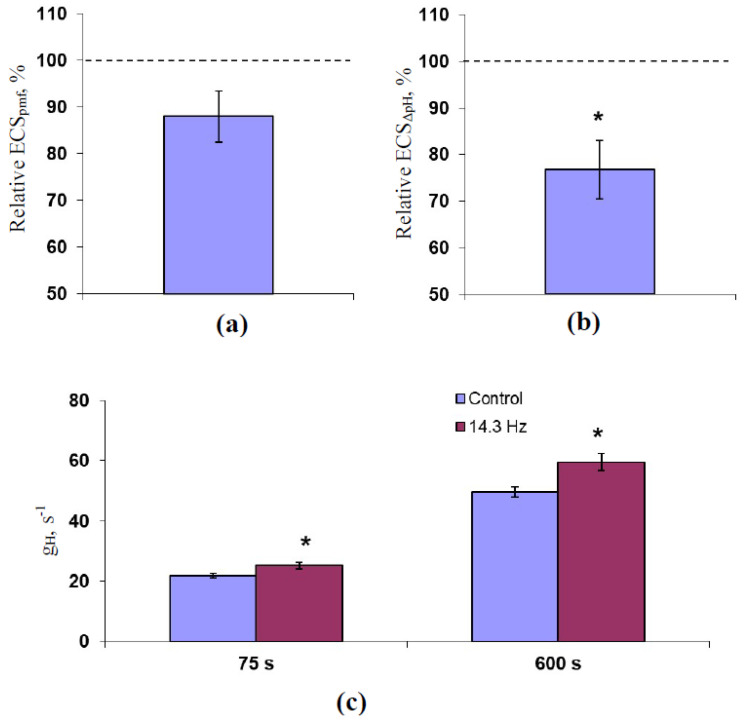
Influence of extremely low-frequency magnetic field (ELFMF) with 14.3 Hz frequency and 18 µT magnitude on relative values of the proton motive force (ECS_pmf_) (**a**), proton gradient (ECS_ΔpH_) (**b**), and H^+^ conductivity (**c**) across the thylakoid membrane (*n* = 6). Measurement of the electrochromic shift (ECS) was initiated after 15 min of dark adaptation. ECS_pmf_ and ECS_ΔpH_ were calculated on basis of different components of ECS after 600 s of the illumination; relative values under the ELFM treatment were calculated as a percentage from the same control values. g_H_ was calculated as the exponential velocity of the ECS relaxation after 75 and 600 s of illumination. The ECS measurement and analysis is described in Section “Materials and Methods” in more detail. The action of the artificial ELFMF was initiated before the dark adaptation; ECS parameters were measured under the treatment. Control seedlings were not treated by the ELFMF. *, the difference between photosynthetic parameters in the experimental and control wheat seedlings was significant (*p* < 0.05).

It was shown that treatment by ELFMF insignificantly decreased ECS_pmf_ (*p* < 0.10, Figure 4a) and significantly decreased ECS_ΔpH_ (Figure 4b) after 600 s of illumination by the actinic light. The g_H_ increased under the treatment by ELFMF with 14.3 Hz frequency after 75 and 600 s of the illumination (Figure 4c).

### 2.3. Development of the Simple Model of H^+^ Fluxes across the Thylakoid Membrane under Illumination

**Figure 5:**
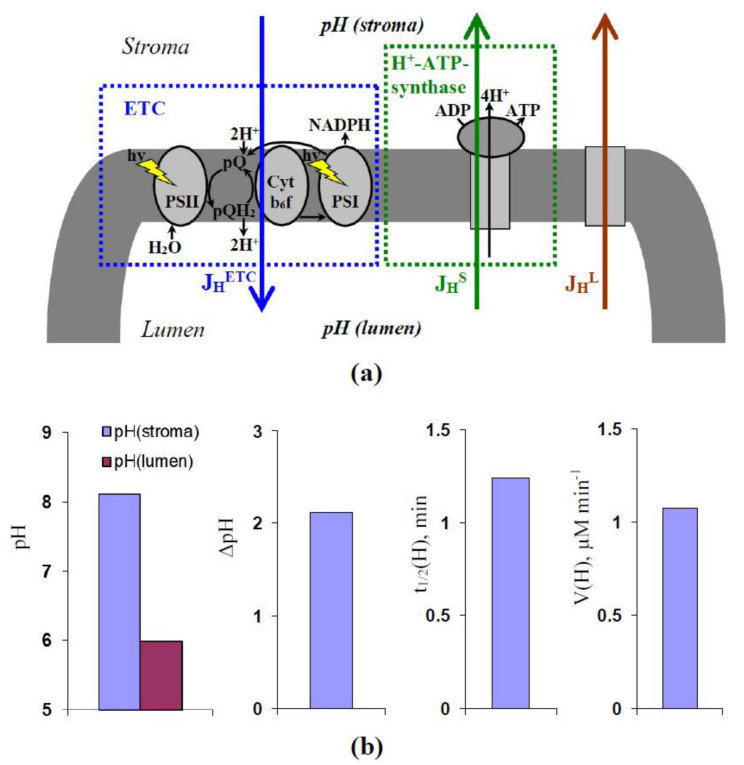
(**a**) Scheme of main H^+^ fluxes described in the model. J_H_^ETC^ is the H^+^ flux per volume caused by the chloroplast electron transport chain (ETC) (Equation (3)). J_H_^S^ is the H^+^ flux per volume caused by the H^+^-ATP synthase (Equation (4)). J_H_^L^ is the additional integral H^+^ flux per volume, which can be caused by proton leakage and/or activity of H^+^ transporters in the thylakoid membrane including the H^+^/Ca^2+^ antiporter, H^+^/K^+^ antiporter, and the proton/phosphate transporters (Equation (5)). PSI and PSII are the photosystems I and II, respectively. Cyt b_6_f is the cytochrome b_6_f complex. pQ and pQH_2_ are the pools of plastoquinone and plastoquinol, respectively; (**b**) parameters of changes in H^+^ concentrations simulated by the model. pH(stroma) and pH(lumen) are the stationary stromal and luminal pH in chloroplasts (Equations (2) and (7)). ΔpH is the pH gradient equaling to the difference between pH(stroma) and pH(lumen). t_1/2_(H) is the time taken for a 50% increase in H^+^ concentration in the lumen of chloroplasts (Equation (8)). V(H) is the absolute velocity of change in H^+^ concentration in the lumen at the initiation of illumination ((Equation (9)). The analysis was carried out at k_ETC_ = 0.05 s^−1^ (the velocity constant of H^+^ transport through ETC), k_S_ = 0.13 s^−1^ (the velocity constant of H^+^ transport through the H^+^-ATP synthase), and k_L_ = 0 s^−1^ (the velocity constant of the additional H^+^ flux).

Considering our hypothesis about the participation of changes in proton fluxes in the effect of the ELFMF treatment, we developed the simplest description of H^+^ fluxes across the thylakoid membrane (Figure 5a).

Equation (1) was used for the description of changes in concentration of protons in the lumen of chloroplasts ([H^+^]_lumen_).

(1) $$\frac{d{\left[H^{+}\right]}_{\mathrm{lumen}}}{\mathrm{dt}}=J_{H}{}^{\mathrm{ETC}}-J_{H}{}^{S}-J_{H}{}^{L}$$

where J_H_^ETC^ is the H^+^ flux per volume (M s^−1^) caused by ETC of chloroplasts [21,22,23,24,25], J_H_^S^ is the H^+^ flux per volume (M s^−1^) caused by the H^+^-ATP synthase [21,23,25], J_H_^L^ is the additional integral H^+^ flux per volume (M s^−1^), which can be caused by proton leakage [11,38,39] and/or activity of H^+^ transporters in the thylakoid membrane including the H^+^/Ca^2+^ antiporter [40], the H^+^/K^+^ antiporter [41,42,43], and the proton/phosphate transporters [44]. It should be noted that we did not include a description of the buffer capacity of the stroma and lumen in the model. We assumed that [H^+^] is the constant fraction of the total proton concentration (α < 1). In this case, J_H_^ETC^, J_H_^S^, and J_H_^L^ can be considered as effective fluxes influencing only [H^+^]; they equal to multiplication of α and total values of these fluxes influencing the total proton concentration. However, this detailed description based on α and these total fluxes is redundant; therefore, it was excluded from the model.

The concentration of protons in the stroma of chloroplasts ([H^+^]_stroma_) was calculated on basis of Equation (2) as follows:

(2) $${\left[H^{+}\right]}_{\mathrm{stroma}}=V_{L}/V_{S}\left(H_{S}-{\left[H^{+}\right]}_{\mathrm{lumen}}\right)$$

where V_L_/V_S_ is the ratio of volumes of the lumen and the stroma (it was assumed as 0.1 because the ratio is 10–17% in accordance with [61]), and H_S_ is the ratio of the total quantity of protons in the stroma and the lumen to the volume of the lumen. H_S_ was calculated as $V_{S}/V_{L}{\left[H^{+}\right]}_{\mathrm{stroma}}{}^{0}+{\left[H^{+}\right]}_{\mathrm{lumen}}{}^{0}$, where [H^+^]_stroma_^0^ and [H^+^]_lumen_^0^ are dark concentrations of protons in the stroma and lumen, respectively. It was accepted that both values are 10^−7^ M because pH in the stroma and the lumen is 7.0–7.5 under dark conditions [61]; therefore, H_S_ equaled to 1.1 × 10^−6^ M.

Descriptions of J_H_^ETC^, J_H_^S^, and J_H_^L^ were based on the equations of chemical kinetics. For simplification of description, we excluded the electrical potential of the thylakoid membrane from the model because this potential is relatively low (the stationary electrical potential of the thylakoid membrane after 1–2 s of illumination is about 20 mV [62,63]).

Equation (3) was used for the description of J_H_^ETC^; the activity of ETC was described as a simple system of proton transport.

(3) $$J_{H}{}^{\mathrm{ETC}}=k_{\mathrm{ETC}}{\left[H^{+}\right]}_{\mathrm{stroma}}$$

where k_ETC_ is the velocity constant of the proton transport by ETC across the thylakoid membrane. There are two important points related to Equation (3). (i) The transport of electron and H^+^ by the ETC accompanies large changes in the redox potentials of components of ETC [24]. Based on this fact, we assumed that the velocity constant of the reverse proton transport by ETC was about zero. (ii) It is known [25] that the proton transport through the pool of plastoquinone (pQ) accompanies an uptake of two protons. It means that Equation (3) can potentially be the bimolecular reaction ($J_{H}{}^{\mathrm{ETC}}=k_{\mathrm{ETC}}{\left[H^{+}\right]}_{\mathrm{lumen}}{}^{2}\;$in this case). However, in accordance with [64], the uptake of the first H^+^ by pQ is a fast process; in contrast, the uptake of the second H^+^ is a slow process. It can be supposed that just uptake of the second H^+^ limits the velocity of the proton transport through ETC. Thus, we assumed that J_H_^ETC^ can be described as a monomolecular reaction (see Equation (3)).

Equation (4) was used for the description of J_H_^S^.

(4) $$J_{H}{}^{S}=k_{S}\left({\left[H^{+}\right]}_{\mathrm{lumen}}e^{\frac{{\Delta G}_{\mathrm{ATP}}}{\mathrm{mRT}}}-{\left[H^{+}\right]}_{\mathrm{stroma}}\right)$$

where k_S_ is the velocity constant of the proton transport by the H^+^-ATP synthase across the thylakoid membrane, ΔG_ATP_ is the energy of hydrolysis of ATP (−50 kJ mol^−1^ [65]), R and T are standard thermodynamic parameters (8.31 J K^−1^ and 295 K, respectively), m is the quantity of H^+^, which is necessary for the synthesis of 1 ATP by the H^+^-ATP synthase (in accordance with [25], m = 3–5; we assumed that m = 4).

Equation (5) was used for the description of J_H_^L^.

(5) $$J_{H}{}^{L}=k_{L}\left({\left[H^{+}\right]}_{\mathrm{lumen}}-{\left[H^{+}\right]}_{\mathrm{stroma}}\right)$$

where k_L_ is the velocity constant of the additional proton transport by proton leakage [11,38,39] and/or activity of H^+^ transporters in the thylakoid membrane [40,41,42,43,44].

Equation (6) describing the dynamics of [H^+^]_lumen_ after the initiations of the illumination was derived from Equations (1)–(5) as follows:

(6) $${\left[H^{+}\right]}_{\mathrm{lumen}}=\frac{\left(k_{\mathrm{ETC}}+k_{S}+k_{L}\right)H_{S}\frac{V_{L}}{V_{S}}}{\left(k_{\mathrm{ETC}}+k_{S}+k_{L}\right)\frac{V_{L}}{V_{S}}+k_{S}e^{\frac{{\Delta G}_{\mathrm{ATP}}}{\mathrm{mRT}}}+k_{L}}\;+\;\left({\left[H^{+}\right]}_{\mathrm{lumen}}{}^{0}-\frac{\left(k_{\mathrm{ETC}}+k_{S}+k_{L}\right)H_{S}\frac{V_{L}}{V_{S}}}{\left(k_{\mathrm{ETC}}+k_{S}+k_{L}\right)\frac{V_{L}}{V_{S}}+k_{S}e^{\frac{{\Delta G}_{\mathrm{ATP}}}{\mathrm{mRT}}}+k_{L}}\right)e^{-\left(\left(k_{\mathrm{ETC}}+k_{S}+k_{L}\right)\frac{V_{L}}{V_{S}}+k_{S}e^{\frac{{\Delta G}_{\mathrm{ATP}}}{\mathrm{mRT}}}+k_{L}\right)t}$$

where t is the time after the initiation of the illumination.

Equation (7) showing the stationary [H^+^]_lumen_ ([H^+^]_lumen_^st^) after the initiation of illumination was derived from Equation (6):

(7) $${\left[H^{+}\right]}_{\mathrm{lumen}}{}^{\mathrm{st}}=\frac{\left(k_{\mathrm{ETC}}+k_{S}+k_{L}\right)H_{S}\frac{V_{L}}{V_{S}}}{\left(k_{\mathrm{ETC}}+k_{S}+k_{L}\right)\frac{V_{L}}{V_{S}}+k_{S}e^{\frac{{\Delta G}_{\mathrm{ATP}}}{\mathrm{mRT}}}+k_{L}}$$

Equation (7) was used for the calculation of the stationary luminal pH (pH(lumen)). Equations (7) and (2) were used for the calculation of the stationary stromal pH (pH(stroma)). The pH gradient across the thylakoid membrane (ΔpH) was calculated as the difference between pH(lumen) and pH(stroma).

Equation (8) shows the time taken for the 50% increase in the H^+^ concentration in the lumen of chloroplasts after the initiation of illumination (t_1/2_(H)). This equation was derived from Equation (6):

(8) $$t_{1/2}\left(H\right)\;=\;\frac{1}{\left(\left(k_{\mathrm{ETC}}+k_{S}+k_{L}\right)\frac{V_{L}}{V_{S}}+k_{S}e^{\frac{{\Delta G}_{\mathrm{ATP}}}{\mathrm{mRT}}}+k_{L}\right)\ln2}$$

Equation (9) shows the absolute velocity of change in H^+^ concentration in the lumen at the initiation of illumination (V(H)). This equation was derived from Equation (6).

(9) $$V\left(H\right)\;=\;-\left(\left(k_{\mathrm{ETC}}+k_{S}+k_{L}\right)\frac{V_{L}}{V_{S}}+k_{S}e^{\frac{{\Delta G}_{\mathrm{ATP}}}{\mathrm{mRT}}}+k_{L}\right)\left({\left[H^{+}\right]}_{\mathrm{lumen}}{}^{0}-{\left[H^{+}\right]}_{\mathrm{lumen}}{}^{\mathrm{st}}\right)$$

The decreased luminal pH causes induction of the energy-dependent component of NPQ [26,27,28,31]; this means that [H^+^]_lumen_^st^ can be strongly related to NPQ_F_. In accordance with [41,42], changes in NPQ can be related to changes in the luminal pH, which means that t_1/2_(H) can be related to t_1/2_(NPQ) and V(H) can be related to V(NPQ).

Further, the question “Can the developed model describe realistic values of pH in the chloroplast?” was analyzed. For simplification of analysis, we assumed that k_L_ = 0 (the additional H^+^ transport was absent); k_ETC_ and k_L_ were varied. It was shown (Figure 5b) that the model with k_ETC_ = 0.05 s^−1^ and k_S_ = 0.13 s^−1^ simulated pH(lumen) and pH(stroma) equaling to about 6 and 8, respectively; ΔpH was about 2. The results were in good accordance with experimental values—(pH(lumen) is 5.7–6.5 and pH(stroma) is about 8 [61]). t_1/2_(H) was 1.24 min that was in accordance with the experimental t_1/2_(NPQ) in the control seedlings (1.23 ± 0.04 min, Figure 3b). V(H) was about 1.07 µM min^−1^.

### 2.4. Theoretical Analysis of the Potential Ways ELFMF Influenced the Parameters of Photosynthetic Light Reactions

**Figure 6:**
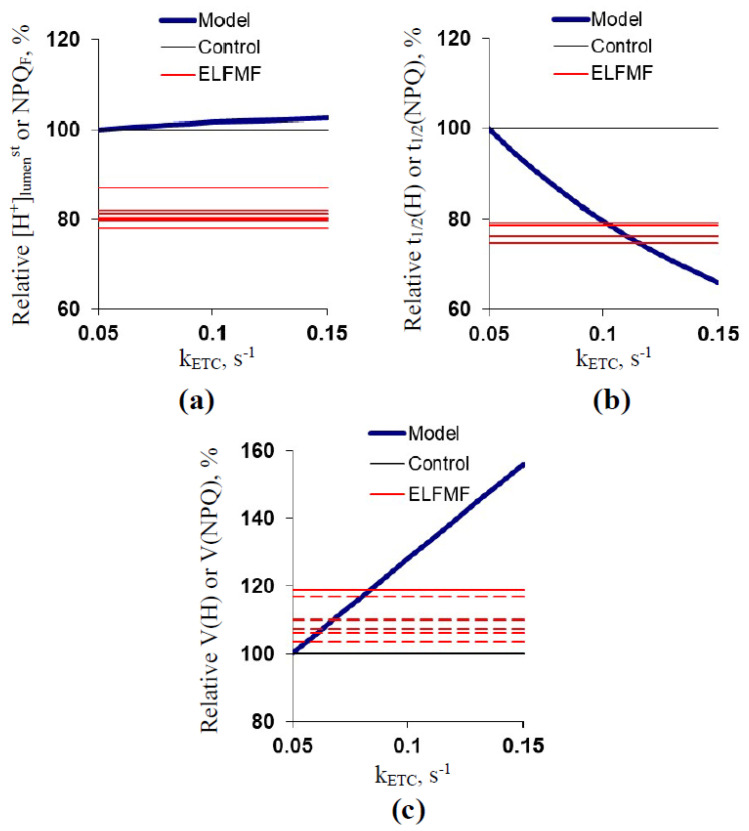
Dependencies of simulated stationary [H^+^]_lumen_ ([H^+^]_lumen_^st^) (**a**), t_1/2_(H) (**b**), and V(H) (**c**) on k_ETC_. Relative values were calculated as percentage from simulated values at k_ETC_ = 0.05 s^−1^, k_S_ = 0.13 s^−1^, and k_L_ = 0 s^−1^ (see Figure 5b). Figure 6 also shows relative control and experimental averaged values of NPQ_F_ (**a**), t_1/2_(NPQ) (**b**), and V(NPQ) (**c**); the experimental values include all variants of ELFMF treatments (with different ELFMF intensities). The results from Figure 2 and Figure 3 were used. Relative values were calculated as a percentage from control values; standard errors were not included in Figure 6. Dotted lines show experimental parameters, which did not significantly differ from the control.

**Figure 7:**
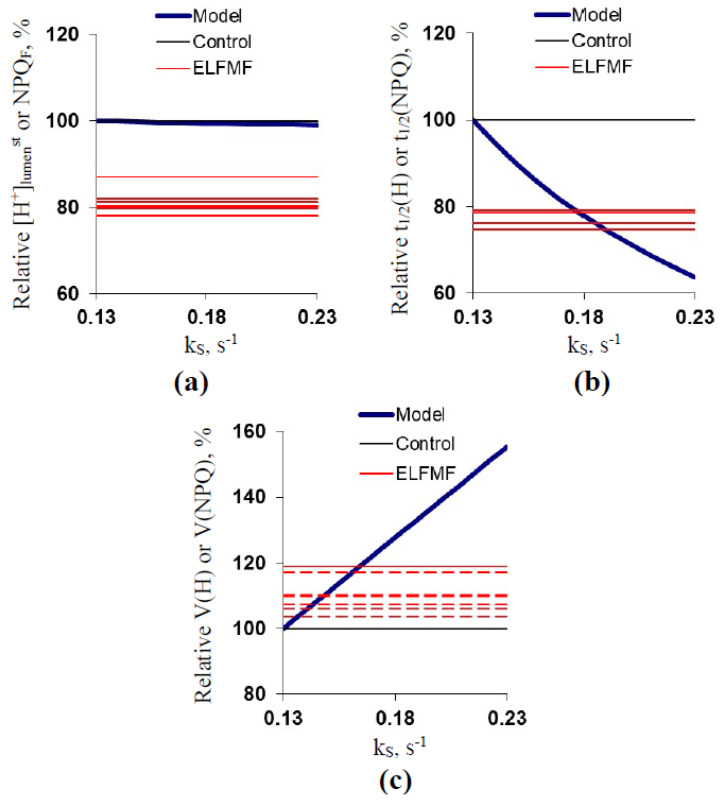
Dependencies of simulated [H^+^]_lumen_^st^ (**a**), t_1/2_(H) (**b**), and V(H) (**c**) on k_S_. Relative values were calculated as percentage from simulated values at k_ETC_ = 0.05 s^−1^, k_S_ = 0.13 s^−1^, and k_L_ = 0 s^−1^ (see Figure 5b). Figure 7 also shows relative control and experimental averaged values of NPQ_F_ (**a**), t_1/2_(NPQ) (**b**), and V(NPQ) (**c**); the experimental values include all variants of ELFMF treatments (with different ELFMF intensities). The results from Figure 2 and Figure 3 were used. Relative values were calculated as a percentage from control values; standard errors were not included in Figure 7. Dotted lines show experimental parameters, which did not significantly differ from the control.

**Figure 8:**
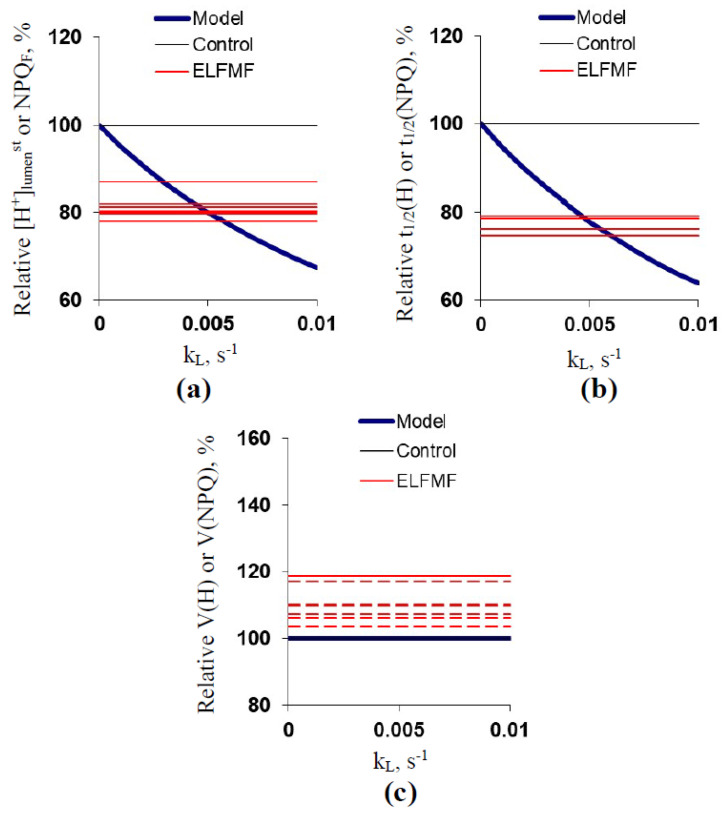
Dependencies of simulated [H^+^]_lumen_^st^ (**a**), t_1/2_(H) (**b**), and V(H) (**c**) on k_L_. Relative values were calculated as percentage from simulated values at k_ETC_ = 0.05 s^−1^, k_S_ = 0.13 s^−1^, and k_L_ = 0 s^−1^ (see Figure 5b). Figure 8 also shows relative control and experimental averaged values of NPQ_F_ (**a**), t_1/2_(NPQ) (**b**), and V(NPQ) (**c**); the experimental values include all variants of ELFMF treatments (with different ELFMF intensities). The results from Figure 2 and Figure 3 were used. Relative values were calculated as a percentage from control values; standard errors were not included in Figure 8. Dotted lines show experimental parameters, which did not significantly differ from the control.

Results of Section 2.3. showed that the developed model can simulate experimental parameters of pH in the stroma and lumen of chloroplasts, which means that this model can be used for the analysis of potential ways ELFMF influences the parameters of photosynthetic light reactions. This analysis was performed in the section (Figure 6, Figure 7 and Figure 8).

Figure 6 shows the dependencies of [H^+^]_lumen_^st^, t_1/2_(H), and V(H) on k_ETC_. The parameters were compared to the NPQ_F_, t_1/2_(NPQ), and V(NPQ) because these parameters should be strongly related to pH(lumen) [26,27,28,31,41,42] and were affected by the 14.3 Hz ELFMF. It was shown that the increase in k_ETC_ weakly increased [H^+^]_lumen_^st^ (Figure 6a); in contrast, the ELFMF treatment induced significant decreases in values of NPQ_F_. The increased k_ETC_ (about 0.10–0.12 s^−1^) induced a decrease in t_1/2_(H) with a magnitude equaling to magnitudes of decreases in t_1/2_(NPQ) under the ELFMF treatment (Figure 6b). V(H) was strongly increased with the k_ETC_ increase (Figure 6c). The last changes were not similar to changes in V(NPQ) because only weak and non-significant changes in this parameter were observed under the ELFMF treatment.

Figure 7 shows the dependencies of [H^+^]_lumen_^st^, t_1/2_(H), and V(H) on k_S_. It was shown that the increase in k_S_ weakly decreased [H^+^]_lumen_^st^ (Figure 7a); however, the magnitude of this decrease was strongly lower than the magnitudes of ELFMF-induced decreases in NPQ_F_. The increased k_S_ (about 0.17–0.19 s^−1^) induced a decrease in t_1/2_(H) with a magnitude equaling to magnitudes of decreases in t_1/2_(NPQ) under the ELFMF treatment (Figure 7b). V(H) was strongly increased with the k_S_ increase (Figure 7c). The last changes were not similar to changes in V(NPQ) because only weak and non-significant changes in this parameter were observed under the ELFMF treatment.

Results (Figure 6 and Figure 7) showed that changes in both k_ETC_ and k_S_ could not cause responses of [H^+^]_lumen_^st^, t_1/2_(H), and V(H) similar to ELFMF-induced responses of NPQ_F_, t_1/2_(NPQ), and V(NPQ). We hypothesized that an increase in the additional H^+^ transport across the thylakoid membrane (proton leakage or H^+^ transport through transporters) could cause responses similar to the ELFMF-induced ones.

Figure 8 shows the dependencies of [H^+^]_lumen_^st^, t_1/2_(H), and V(H) on k_L_. It was shown that an increase in k_L_ strongly decreased [H^+^]_lumen_^st^ (Figure 8a); the magnitude of this decrease was similar to magnitudes of ELFMF-induced decreases in NPQ_F_ at k_L_ = 0.003–0.006 s^−1^. The increased k_L_ (about 0.005–0.006 s^−1^) also induced a decrease in t_1/2_(H) with magnitude equaling to magnitudes of the decreases in t_1/2_(NPQ) under the ELFMF treatment (Figure 8b). Finally, the increase in k_L_ did not influence V(H) (Figure 8c). The last result was rather in accordance with experimental data because only weak and non-significant changes in V(NPQ) were observed under the ELFMF treatment.

## 3. Discussion

It is well known that photosynthesis can be affected by numerous environmental factors including physical factors (e.g., non-optimal temperatures [12,13,14,15,16,17], high-intensity visible light [1,2,3,4,5], or ultraviolet light [6]); however, the potential influence of ELFMFs on photosynthetic processes is still weakly investigated. There are few works [52,54] which showed that treatment of plants by ELFMFs with the 50 Hz industry frequency can modify the photosynthetic CO_2_ assimilation. Earlier, it was shown [57] that the short-term and chronic treatment by ELFMFs with frequencies of the Schumann resonance (7.8, 14.3, and 20.8 Hz) and the 18 µT intensity influenced parameters of photosynthetic light reactions. This treatment decreased NPQ_max_, NPQ_F_, NPQ_S_, t_1/2_(Φ_PSII_), and t_1/2_(NPQ) in wheat seedlings [57]; the effect was strongest at the treatment by the 14.3 Hz ELFMF. In the current work, we analyzed the ELFMF influence on photosynthetic light reactions in more detail, which revealed three groups of results.

First, the short-term treatment by the 14.3 Hz ELFMF decreased NPQ_max_, NPQ_F_, NPQ_S_, t_1/2_(Φ_PSII_), and t_1/2_(NPQ) in wheat seedling in a wide range of magnitudes of this magnetic field (from 3 to 180 µT). This result supports the potential importance of the revealed effect for plants. It is interesting that the 14.3 Hz ELFMF could also increase Φ_PSII_^L^; however, significant changes were observed at certain intensities of these magnetic fields. In contrast, the treatment by the 14.3 Hz ELFMF weakly increased V(NPQ) and did not influence F_v_/F_m_ and t_1/2_(NPQ relaxation).

Considering the results, the following potential ways of the ELFMF influence on photosynthetic light reactions can be discussed: (i) absence of effect of the 14.3 Hz ELFMF on the potential quantum yield of PSII showed that strong influence of this ELFMF on initial electric charge separation was not likely because F_v_/F_m_ should be sensitive to large changes in this process [66]; (ii) absence of changes in t_1/2_(NPQ relaxation) excluded stimulation of de-protonation of PsbS proteins (mechanism of the relaxation of the energy-dependent component of NPQ [4,31]) by the ELFMF treatment. It is interesting that the ELFMF-induced stimulation of protonation of the PsbS proteins was also improbable because this stimulation should increase NPQ_F_ [4,31,67,68]. Additionally, the result rather excluded participation of ELFMF-induced changes in the activity of the H^+^/K^+^ antiporter of the thylakoid membrane [41,42,43]. It is known [42] that this antiporter is not active under high-intensity illumination and can be activated after a decrease in the light intensity. If the 14.3 Hz ELFMF modified the activity of the H^+^/K^+^ antiporter then we should have observed changes in the NPQ dark relaxation and should not have observed the changes in the light-induced NPQ increase; however, the opposite result was shown; (iii) the main ELFMF-induced changes were observed in values of parameters of NPQ, which was strongly related to the luminal pH [4,31,67,68], in Φ_PSII_^L^, which should be also affected by this pH [22,69], and in t_1/2_(Φ_PSII_), which could be related to the stromal pH-induced activation of the photosynthetic dark reactions [33,34,35] and changes in the localization of the ferredoxin–NADP reductase [36,37]. These results support our preliminary hypothesis about H^+^ fluxes as the potential target of the ELFMF action [57].

Second, analysis of ECS, which can be effectively used for estimation of proton and electrical gradients across the thylakoid membrane [58,59,60], showed that the treatment by 14.3 Hz ELFMF insignificantly decreased the total value of this shift, which is related to the proton motive force, and significantly decreased its component, which is related to the proton gradient. These results were in good accordance with the ELFMF-induced decreasing NPQ_F_, which is also related to pH in lumen and proton gradient across the thylakoid membrane [4,31,67,68]. The finding that 14.3 Hz ELFMF-induced changes increased the exponential velocity of the dark relaxation of ECS, which is strongly related to the proton conductivity across the thylakoid membrane [59,60], additionally supports the hypothesis of the participation of changes in H^+^ fluxes in the modification of photosynthetic processes by this magnetic field. It is important that g_H_, which is measured under dark conditions [59], could not be related to H^+^ fluxes induced by the activity of ETC. This means that 14.3 Hz ELFMF should modify the activity of the H^+^-ATP synthase and/or additional H^+^ fluxes across the thylakoid membrane.

Third, the theoretical analysis showed that the H^+^ fluxes through ETC and the H^+^-ATP synthase were not the probable target of influence of the 14.3 Hz ELFMF. Stimulation of these fluxes could induce the decrease in t_1/2_(H), which corresponded with the decrease in t_1/2_(NPQ); however, the weak changes in [H^+^]_lumen_^st^, which corresponded with NPQ_F,_ and the strong increase in V(H), which corresponded with V(NPQ), were not in accordance with experimental changes. In contrast, the increase in the additional H^+^ flux decreased [H^+^]_lumen_^st^ and t_1/2_(H) and did not influence V(H). The theoretical results were in good accordance with experimental ones; it means that stimulation of this additional H^+^ flux is a probable mechanism of the FLFMF influence on photosynthetic light reactions. It should be noted that g_H_ and constants of the model (k_S_ and k_L_) strongly differed. This is not contradictory to our hypothesis because g_H_ is related to the total flux of charge across the thylakoid membrane, and k_S_ and k_L_ are related to the effective proton fluxes (see Section 2.3.), which can be strongly lower than these charge fluxes.

However, there were different potential ways of this stimulation because the additional H^+^ flux includes the passive proton leakage across the thylakoid membrane [11,38,39] and the H^+^ transport by transporters including the H^+^/Ca^2+^ antiporter [40], the H^+^/K^+^ antiporter [41,42,43], and the proton/phosphate transporters [44]. As noted above, participation of H^+^/K^+^ antiporter in the thylakoid membrane in the revealed effect did not seem to be probable. The possibility of participation of the proton/phosphate transporters in the ELFMF influence is not clear.

**Figure 9:**
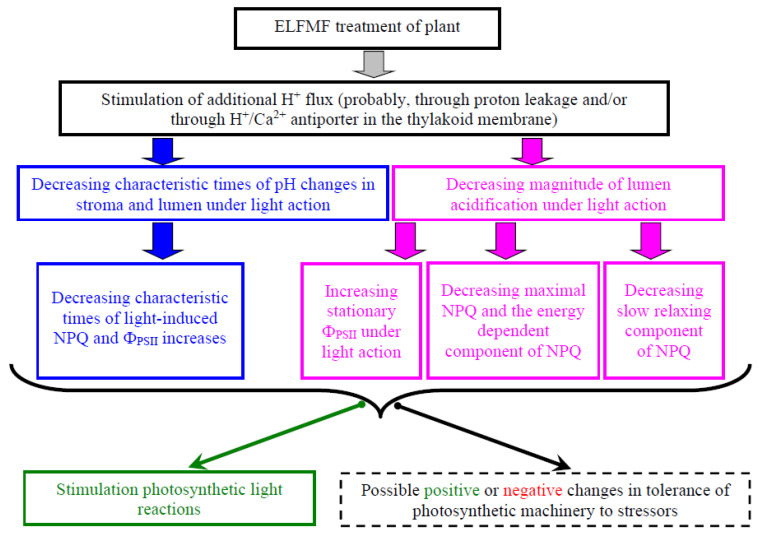
Scheme of potential ways of influence of the 14.3 Hz extremely low-frequency magnetic fields (ELFMF) on photosynthetic parameters of plants (see Section 3 for details). The dotted box shows the possible influence of ELFMF on photosynthetic tolerance to actions of stressors; however, this influence requires further research.

In contrast, it is known [70] that ELFMFs can increase the membrane permeability for ions (including protons); the effect was shown for 10 and 100 µT intensities and 50 and 60 Hz frequencies of the magnetic fields. The result showed that the ELFMF-induced stimulation of the proton leakage across the thylakoid membrane could be a potential mechanism of the revealed changes in photosynthetic light reactions in wheat. The alternative mechanism can be related to increased free Ca^2+^ concentration in the stroma and lumen because ELFMFs can influence Ca^2+^ homeostasis in plants and increase the free Ca^2+^ concentration [71,72,73,74,75] that can be related to direct or indirect effects by the cyclotron resonance [48,74]. The increased concentration of Ca^2+^ can stimulate the H^+^/Ca^2+^ antiporter in the thylakoid membrane, i.e., it can stimulate the additional H^+^ flux across this membrane. Thus, the results of the current work showed potential ways of influence of ELFMF with the Schumann resonance frequencies (at least 14.3 Hz) on photosynthetic light reactions (Figure 9).

Considering the decreased NPQ and the increased Φ_PSII_ after the ELFMF treatment (results of the current work and previous work [57]), it can be concluded that this treatment rather stimulated photosynthetic light reactions and perhaps increased the plant productivity. Stimulation of the plant growth and the production of biomass by ELFMF treatments [48,49,50,51] were in accordance with this effect. The acceleration of the light-induced increase in Φ_PSII_ (the decrease in t_1/2_(Φ_PSII_), which was shown in the current work and in [57]), should also contribute to the stimulation of photosynthetic light reactions.

Changes in tolerance of plants to stressors (particularly, the tolerance of photosynthetic machinery) can be considered as an expected result of the ELFMF-induced changes in photosynthetic processes. However, the potential influence of the ELFMF treatment on tolerance of the photosynthetic machinery to actions of stressors seems to be contradictory. The ELFMF-induced decrease in NPQ_S_, which can be related to the photodamage [4,27,28], showed that this MF probably increase the photosynthetic tolerance to the excess light. In contrast, a decrease in NPQ_F_, which is traditionally considered as the mechanism of photosynthetic machinery protection [4,26,31], could decrease the tolerance of the photosynthetic to actions of stressors. Moreover, the decrease in NPQ_F_ and the increase in Φ_PSII_^L^ can contribute to the light-induced production of the reactive oxygen species [76,77,78], which participate in both the damage of cell structures and the stress signaling triggering the adaptation changes. The influence of ELFMFs on the tolerance of the photosynthetic machinery requires further experimental and theoretical investigations.

Thus, the results of this investigation clarified the potential ways of influence of ELFMF on photosynthetic light reactions; these results may be important for investigations in the field of plant physiology, plant ecology, and plant cultivation. Revealing specific systems of additional proton transport participating in the ELFMF-induced photosynthetic changes, analysis of relations of the ELFMF effects to plant productivity and tolerance, analysis of the potential influence of these fields on photosynthetic signaling (e.g., production of reactive oxygen species), investigation of possibility of additional influence of the ELFMF on primary electron transport, and studying differences in the ELFMF-induced photosynthetic changes in different species of plants are important tasks of future investigations. The last point is especially interesting because a study [57] showed that ELFMF did not influence photosynthetic light reactions in pea seedlings; it cannot be excluded that the difference in these photosynthetic responses will be also observed in the other plant species. Considering Equations (7)–(9), it can be expected that the influence of changes in the additional H^+^ fluxes should be low at high k_ETC_ and k_S_ and vice versa. Values of these effective velocity constants are dependent on the content of ETC and H^+^-ATP synthase in the thylakoid membrane, area/volume ratio (k_ETC_ and k_S_ are calculated per volume), parameters of photosynthetic light regulation, etc.; these parameters can be varied in different plant species, which means that differences in sensitivity to ELFMF are probable for different plant species.

## 4. Materials and Methods

### 4.1. Materials

Wheat seedlings (*Triticum aestivum* L., cultivar Zlata) were used in experiments. Seeds were soaked for 2 days before plant planting. Plants were cultivated (up to 12–13 days age) in vegetation pots with the standard soil (universal soil “Dobrii pomoshnik”, Morris Green) in open ground conditions (duration of the light day was about 17.6 h, the average light intensity for the day was about 94 ± 19 µmol m^−2^s^−1^, averaged day and night temperatures were 27 ± 1 and 18 ± 1 °C) with regular irrigation. Positions of control and experimental vegetation pots were randomized at cultivation.

### 4.2. Treatments by the 14.3 Hz ELFMF and Photosynthetic Measurements

The general design of the experiment was similar to the design used in a previous study on short-term treatment by ELFMF with Schumann resonance frequencies [57]; however, different intensities of the 14.3 Hz ELFMF were investigated.

**Figure 10:**
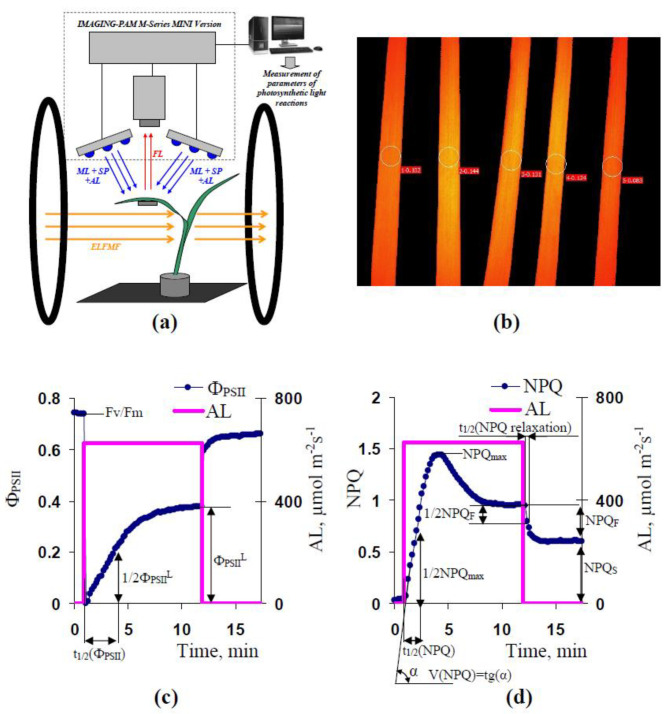
(**a**) Schema of plant localization in experiments with measurements of parameters of photosynthetic light reactions with using IMAGING-PAM M-Series MINI Version under simultaneous treatment by the artificial extremely low-frequency magnetic field (ELFMF). AL is actinic light, ML is measuring light, and SP is saturation pulse. Blue light (450 nm) was used for the illumination of leaves. FL is chlorophyll fluorescence; (**b**) localization of investigated areas (ROIs) at PAM-imaging in wheat leaves; (**c**) change in quantum yield of photosystem II (Φ_PSII_) under AL action and estimation of parameters of this change. F_v_/F_m_ is the potential quantum yield of photosystem II, Φ_PSII_^L^ is the effective quantum yield of photosystem II (PSII) after 10 min of illumination by AL, and t_1/2_(Φ_PSII_) is the time of 50% increase in Φ_PSII_ under illumination; (**d**) change in non-photochemical quenching (NPQ) under the AL action. NPQ_F_ is the fast-relaxing component of NPQ after 10 min of illumination, NPQ_S_ is the slow-relaxing component of NPQ after this illumination, NPQ_max_ is the maximal value of NPQ, t_1/2_(NPQ) is the time of 50% increase in NPQ under illumination, V(NPQ) is the initial linear velocity of the NPQ increase, and t_1/2_(NPQ relaxation) is the time of 50% decrease in NPQ_F_ after the termination of AL.

The manufactured system for the treatment of plants by ELFMF (volume of the homogenous magnetic field was about 20 × 20 × 20 cm^3^) at simultaneous measurements of parameters of photosynthetic light reactions was used in the investigation (Figure 10a). This system was based on Helmholtz coils (100 loops) with a 0.3 m radius. Positions of Helmholtz coils supported the direction of ELFMFs, which was perpendicular to the direction of the geomagnetic field (about 50 µT). RIGOL DG1032 Waveform Generator (RIGOL Technology Co., Ltd., Suzhou, China) was used for the generation of the sinusoidal electrical signal with a frequency equaling 14.3 Hz. Magnitudes of ELFMFs were 3, 9, 18, 54, 90, 135, and 180 µT.

The treatment of wheat seedlings by the 14.3 Hz ELFMF was initiated after fixation of plants in the system and continued for all the time of the measurement of parameters of photosynthetic light reactions (30 min). In the control variant, wheat seedlings were fixed in this system, and parameters of photosynthetic light reactions were measured; however, the treatment by the artificial ELFMF was absent.

### 4.3. Measurements of Parameters of Photosynthetic Light Reactions

Parameters of photosynthetic light reactions were investigated in all wheat seedlings (excluding control seedlings) simultaneously with the treatment of plants by the 14.3 Hz ELFMF. The total duration of photosynthetic measurements was about 30 min.

A system of pulse–amplitude–modulation (PAM) fluorescence imaging (IMAGING-PAM M-Series MINI Version, Heinz Walz GmbH, Effeltrich, Germany) was used for photosynthetic investigation (Figure 10a). Saturation pulses (SP) with 800 ms duration and 6000 µmol m^−2^s^−1^ intensity, pulses of measuring light (ML) with low average intensity (<1 µmol m^−2^s^−1^), and actinic light (AL) with 625 µmol m^−2^s^−1^ intensity were used in the analysis. Blue light (450 nm) was used for SP, ML, and AL.

Parameters of photosynthetic light reactions were analyzed in the second mature leaves of wheat seedlings. Five wheat leaves from different plants were simultaneously investigated in each experiment (Figure 10b). Photosynthetic parameters were calculated in ROIs placed in the center of each leaf.

The duration of preliminary dark adaptation of wheat leaves was 15 min after leaf fixation in this measuring system. The initial (F_0_) and maximum (F_m_) rates of PSII fluorescence were measured after the dark adaptation at the first SP. After that, SPs were periodically generated every 10 s. The current rate of fluorescence (F) and maximum fluorescence rate under light conditions (F_m_’) were measured at each SP. AL was turned on 80 s after the first SP; the duration of the AL illumination was about 10 min. Periodical SPs were generated for 5 min after termination of AL action.

Using standard equations [27,29,30] F_v_/F_m_, Φ_PSII_, and NPQ were calculated on basis of F_0_, F_m_, F, and F_m_’ by software of IMAGING-PAM. Figure 10c shows the estimation of Φ_PSII_^L^ and t_1/2_(Φ_PSII_). Figure 10d shows the estimation of NPQ_max_, NPQ_F_, NPQ_S_, t_1/2_(NPQ), V(NPQ), and t_1/2_(NPQ relaxation).

### 4.4. Measurement and Analysis of Electrochromic Shift

ECS denotes changes in light absorption with a maximum at 515–520 nm [58], which is strongly related to the electrical gradient across the thylakoid membrane. Curves of the dark relaxation of ECS can be used for revealing the proton motive force, proton gradient, electrical gradient, and H^+^ conductivity across the thylakoid membrane [58,59,60]. Dual-PAM-100 with P515/535 emitter–detector modules (Heinz Walz GmbH, Effeltrich, Germany) was used for measurements of ECS.

Measurement of ECS was initiated after 15 min of the dark adaptation. The intensity of red AL (630 nm) was 660 µmol m^−2^s^−1^. This light was firstly turned off after 75 s of initiation of illumination by AL for estimation of g_H_ (duration of the dark interval was 1 s) and was secondly turned off after 600 s of this initiation for estimation of ECS_pmf_, ECS_ΔpH_, and g_H_ (duration of the dark interval was 60 s; the experiment was terminated after that).

**Figure 11:**
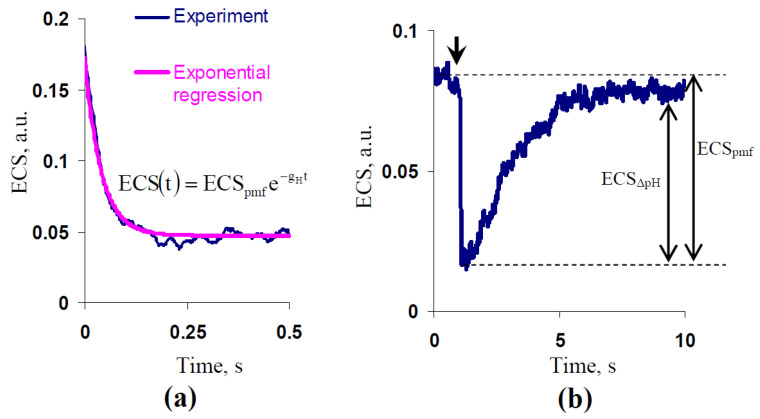
(**a**) Schema of measurement of g_H_, which was calculated as the exponential velocity of the dark relaxation of ECS. The zero point shows the termination of illumination by AL; (**b**) schema of measurements of ECS_pmf_, ECS_ΔpH,_ and ECS_Δψ_. The arrow shows the termination of illumination by AL.

In accordance with [59,60], g_H_ was calculated as the exponential velocity of the dark relaxation of ECS for 500 ms (Figure 11a). In accordance with [58], ECS_pmf_ was calculated as the magnitude of the fast decrease in ECS after the termination of illumination (hundreds of milliseconds), and ECS_ΔpH_ was calculated as the magnitude of following the slow increase in ECS (more than tens of seconds) (Figure 11b). Treatment of ELFMF with 14.3 Hz frequency and 18 µT magnitude was initiated before the initiation of the dark adaptation; it was terminated after the termination of the ECS record. Wheat seedlings were not treated by ELFMF in the control. Relative experimental values of ECS_pmf_ and ECS_ΔpH_ (percentage from the control values) were analyzed.

### 4.5. Statistics

In total, 20–30 wheat seedlings were used for each variant of the experiment (control, 3, 9, 18, 54, 90, 135, and 180 µT). Mean values and standard errors were presented. Student’s *t*-test was used for estimation of the significance of differences between plants treated by ELFMFs and control plants.

## 5. Conclusions

Natural and artificial extremely low-frequency magnetic fields are factors influencing physiological processes in plants. Photosynthetic processes are the important potential target of ELFMF influence. It was experimentally shown that the 14.3 Hz ELFMF (the second harmonic of the Schumann resonance frequencies) with a wide range of magnitudes (3–180 µ) induced a decrease in parameters of the non-photochemical quenching, an increase in stationary effective quantum yield of PSII, and acceleration of light-induced activation of NPQ and Φ_PSII_ in wheat seedlings. In contrast, the maximal quantum yield of PSII and time of the dark relaxation of NPQ_F_ were not influenced by the 14.3 Hz ELFMF. Experimental analysis showed that the influence of ELFMF on photosynthetic light reactions could be related to proton fluxes across the thylakoid membrane because the treatment by this magnetic field induced a decrease in the proton gradient and an increase in H^+^ conductivity across this membrane. The simplest model of H^+^ fluxes through the thylakoid membrane was developed and used for the analysis of ways of influence of ELFMF on photosynthetic light reactions. It was theoretically shown that H^+^ fluxes through the photosynthetic electron transport chain and through H^+^-ATP synthase were not probable targets of action of the 14.3 Hz ELFMF. In contrast, additional H^+^ flux, which can be related to proton leakage and/or H^+^/Ca^2+^ antiporter, seemed to be this potential target of the ELFMF action.

Thus, results of the current work showed that changes in the proton transport across the thylakoid membrane (direct or indirect) can be a potential mechanism of influence of ELFMFs with the Schumann resonance frequencies on photosynthetic light reactions.

## Data Availability

The data presented in this study are available on request from the corresponding author.
